# Skin Wound Healing Following Injecting Hyaluronic Acid Rejuvenating Complex, Polycaprolactone, or Combination Therapy: An Experimental Study

**DOI:** 10.1111/jocd.70221

**Published:** 2025-05-14

**Authors:** Noury Adel, Nenad Stankovic, Gerardo Cervantes, Amira Gindi, Lamiaa Mohamed Shawky

**Affiliations:** ^1^ Oral and Maxillofacial Surgery Specialist, Private Practice Cairo Egypt; ^2^ Doctor of Dental Surgery, Specialist of Cosmetology, Aesthetic Physician Private Practice Belgrade Serbia; ^3^ Aesthetic Physician, Private Practice Valencia Spain; ^4^ Registered Nurse Practitioner Toronto Canada; ^5^ Assistant professor of histology and cell biology, Department of Histology and Cell Biology, Benha Faculty of Medicine Benha University Benha Egypt

**Keywords:** CELLBOOSTER LIFT, ELLANSE, epithelial thickness, Hyaluronic acid, PCL, histological assessment, hyaluronic acid rejuvenating complex, Regenerative medicine, polycaprolactone, skin wound healing

## Abstract

**Purpose:**

This study aimed to investigate the effects of hyaluronic acid rejuvenating complex, polycaprolactone, and their combination on skin wound healing, assessing their potential to accelerate tissue regeneration and optimize healing outcomes.

**Materials and Methods:**

Forty eight Wistar Albino rats were randomly divided into four groups. Group 1 received hyaluronic acid rejuvenating complex injection, Group 2 received polycaprolactone injection, and Group 3 received a combination of both. Group 4 served as the control, undergoing incision without intervention. Skin biopsies were collected at baseline, day 7, and day 14 postincision. Wound healing was evaluated histologically using hematoxylin and eosin (H&E) and Masson's trichrome staining, focusing on epithelial thickness, collagen synthesis, and inflammatory cell infiltration.

**Results:**

The combination therapy group (Group 3) exhibited the most pronounced wound healing response, demonstrating significantly accelerated re‐epithelialization, enhanced collagen deposition, and well‐structured granulation tissue by days 7 and 14. Additionally, inflammatory cell infiltration was markedly reduced, indicating a faster transition from the inflammatory to the proliferative phase. Compared to single‐agent treatments, the combined approach resulted in superior tissue remodeling and a more efficient healing process.

**Conclusion:**

The dual administration of hyaluronic acid rejuvenating complex and polycaprolactone offers a synergistic effect, significantly enhancing skin wound healing compared to monotherapies. These findings highlight the potential of combination therapy as a promising strategy for improving wound repair and tissue regeneration in aesthetic and regenerative dermatology.

## 1 Introduction

Most people consider the body's ability to repair wounds on its own to be a given. However, it is this seemingly unimportant function of the organism that guarantees life from common wounds like cuts and abrasions. Many people do not understand what normal wound healing is until problems like chronic wounds or delayed healing occur. This holds true for the healing processes of every other organ in the body in addition to the compromised integrity of the skin. There has always been strong evolutionary pressure to maximize the effective healing of skin wounds because the skin serves as the most crucial barrier between the sterile interior of the body and the pathogen‐rich external environment [1].

The body's natural response to tissue damage is wound healing. However, the vascular system, cytokines, mediators, and a variety of cell types interact intricately during wound healing, making it a complex process. The initial cascade of platelet aggregation and blood vessel vasoconstriction is intended to halt bleeding. A flood of different inflammatory cells, beginning with the neutrophil, follows. A range of mediators and cytokines are then released by these inflammatory cells to encourage thrombosis, angiogenesis, and reepithelialization. In turn, the fibroblasts deposit extracellular materials that will act as scaffolding. Hemostasis, chemotaxis, and enhanced vascular permeability are characteristics of the inflammatory phase that prevent additional damage, seal the wound, eliminate pathogens and cell debris, and promote cellular migration. The inflammatory stage typically lasts for a few days. Granulation tissue development, reepithelialization, and neovascularization are characteristics of the proliferative phase. This stage may continue for a few weeks. The wound reaches its maximum strength during the maturation and remodeling period [2, 3, 4, 5].

Several nonsurgical modalities have been advocated to accelerate wound healing, those of which include topical formulations, hydrogel‐based grafts, platelet‐rich plasma or fibrin placement, and drug administration or injection [1].

Hyaluronic acid (HA) consists of alternating units of the repeating disaccharide β‐1, 4‐D‐glucuronic acid‐β‐1, 3‐N‐acetyl‐D‐glucosamine, which is broadly found throughout the body and serves as a crucial element of the extracellular matrix (ECM) [6]. Hyaluronic acid is essential in the wound healing process. The growing interest in HA products stems from its diverse biological activities and various physiological functions. HA has the capability to maintain a moist environment that facilitates healing and encourages the proliferation of growth factors, fibroblasts, and keratinocytes. Its highly hydrophilic nature allows HA to absorb exudate and improve cell migration. It has positive effects on wound healing, leading to reduced inflammation, better tissue remodeling, and an enhancement in angiogenesis. HA exerts its biological effects through interactions with specific receptors, such as CD44 and RHAMM (Receptor for Hyaluronan‐Mediated Motility), which influence critical cellular functions, including inflammation control, fibroblast activation, and angiogenesis.

During the early inflammatory phase of wound healing, HA interacts with CD44 receptors on immune cells, such as macrophages and neutrophils, guiding their movement to the wound site. This process facilitates the release of inflammatory mediators that are essential for pathogen clearance and tissue repair. However, HA also plays a role in dampening excessive inflammation by shifting macrophages toward a reparative phenotype, thereby creating an environment conducive to healing. As the wound progresses into the proliferative phase, HA supports fibroblast migration and enhances collagen synthesis, which are crucial for granulation tissue formation. This is mediated by intracellular signaling pathways that promote cell survival and ECM deposition. Additionally, HA contributes to angiogenesis by stimulating endothelial cell activity, ensuring adequate blood supply to the regenerating tissue. Its ability to retain moisture further supports cellular proliferation and migration, expediting wound closure [7].

In the remodeling phase, HA facilitates epithelial cell proliferation and differentiation, leading to efficient re‐epithelialization. It also regulates matrix turnover by modulating the activity of enzymes involved in ECM remodeling, ensuring that the newly formed tissue acquires proper structure and function. Given its multifaceted role in wound healing, HA has become a key focus in regenerative medicine and dermatology. Its ability to regulate inflammation, promote fibroblast activity, and enhance vascularization makes it a promising therapeutic agent for optimizing skin repair [6, 7].

A bioresorbable polymer with collagen‐stimulating qualities is polycaprolactone (PCL). This polymer belongs to the family of aliphatic polyesters, and unlike polylactic acid or polyglycolic acid, which are also aliphatic polyesters, polycaprolactone degrades more slowly. Particularly noteworthy is PCL's adaptability in adding various substituents to its backbone. Tissue engineering scaffolds, artificial blood vessels, wound dressings, neuron regeneration devices, and drug delivery devices are just a few of the numerous therapeutic uses for PCL. PCL is an excellent option for controlled drug delivery applications because of its excellent mechanical qualities and high permeability to numerous medications. PCL's capacity to adjust its chemical and physical characteristics is one of its main benefits over other aliphatic polyesters. By adding the appropriate substituents to their monomers, this is accomplished. By doing this, we may modify PCL's characteristics to our benefit and use them to precisely regulate how drug molecules load and release from PCL‐based drug delivery devices [8].

One of the methods of PCL preparation is as microspheres suspended in a gel carrier for use as a dermal filler. This gel is a completely bioabsorbable nonpermanent dermal filler that is aseptic, free of latex and pyrogen [9]. Several researches evaluated the use of polycaprolactone as a membrane or a nanofibrous scaffold in wound healing and were found to be effective in accelerating the wound healing process, but none of those researches tested the effect of injectable polycaprolactone gel in skin wound healing [10, 11, 12, 13, 14, 15].

That is why this study was important to conduct to test the effect of injectable PCl as well as injectable hyaluronic acid in addition to the combined approach of injecting both products at the same time to accelerate wound healing.

## 2 Materials and Methods

This study was conducted on forty eight adult Wistar albino rats ( *Rattus norvegicus* ) aged 4 to 6 months and weighing 110–170 g. To ensure physiological adaptation and reduce stress‐induced variability, the animals underwent a one‐week acclimatization period before the start of the experiment. The rats were housed in standard ventilated cages in small groups (4 per cage) under a 12‐hour light/dark cycle (lights on at 7:00 AM and off at 7:00 PM) to regulate circadian rhythms and melatonin secretion, which may influence oral wound healing. Environmental conditions, including temperature and humidity, were maintained at optimal laboratory settings. To minimize mechanical irritation to the oral wounds and promote optimal healing, the rats were provided with a soft, nutritionally balanced diet postoperatively. The diet was designed to ensure adequate protein and caloric intake while reducing mechanical stress on the healing tissues. Fresh food and water were replenished daily, and dietary intake was monitored to maintain consistency across all experimental groups. This study was conducted in accordance with institutional ethical guidelines and complied with the principles of the Declaration of Helsinki. Following general anesthesia, all rats were randomly assigned to one of four groups.

Group (1): A no. 15 blade was used to make a 1‐cm incision at the ventral dorsal surface of the hands of the rat, followed by a hyaluronic acid rejuvenating complex injection in the wound bed.

Group (2): A no. 15 blade was used to make a 1‐cm incision at the ventral dorsal surface of the hands of the rat followed by polycaprolactone injection in the wound bed.

Group (3): A no. 15 blade was used to make a 1‐cm incision at the ventral dorsal surface of the hands of the rat followed by injecting both hyaluronic acid rejuvenating complex and polycaprolactone in the wound bed together at the same session.

Group (4): A no. 15 blade was used to make a 1‐cm incision at the ventral dorsal surface of the hands of the rat with no further intervention. (Control group).

The hyaluronic acid rejuvenating complex utilized in this research was a vial that contained sodium hyaluronate, amino acids such as arginine, glycine, proline, valine, lysine, and vitamins such as riboflavin, biotin, sodium ascorbyl phosphate, and tocopherol (CELLBOOSTER LIFT, Suisselle SA, Switzerland). On the other hand, the polycaprolactone product in this study was a prefilled syringe with a white gel that contained PCL spheres dispersed in gel (Ellanse S, Sinclair Pharma; London, United Kingdom).

The dosage of each injectable compound was standardized across all experimental groups. Each rat in the treatment groups received 0.05 ml of hyaluronic acid rejuvenating complex (CELLBOOSTER LIFT, Suisselle SA, Switzerland) and/or 0.05 ml of polycaprolactone‐based dermal filler (Ellansé S, Sinclair Pharma, UK) directly into the wound bed. The selected doses were determined empirically by the research team, based on preliminary observations and the rationale of evaluating their potential efficacy in tissue regeneration, given the absence of prior studies utilizing similar biomaterials. All injections were administered using a BD insulin syringe to ensure precise delivery and avoid excessive tissue trauma; except for Ellansé, it was injected with the needle that comes with the syringe inside the kit.

No postoperative dressings were used on any of the wounds, nor was any antibiotic ointment applied or suturing performed. A biopsy was conducted on an equal number of rats under general anesthesia in each time interval from each group; Biopsy specimens were collected at three timepoints:

– Baseline (Immediately postincision and injection)
– Day 7 (Proliferative phase assessment)
– Day 14 (Early remodeling phase assessment).
– Rats were euthanized at each timepoint using intraperitoneal injection of sodium pentobarbital (200 mg/kg), following ethical guidelines. The wound area was excised with a 3 mm margin surrounding the incision site to ensure a representative tissue sample. The isolated tissue was immediately fixed in 10% neutral‐buffered formalin for 24 h, followed by paraffin embedding for histological sectioning.
– The histological analysis of wound healing was carried out using Hematoxylin and eosin (H&E) staining and Masson trichrome (MT) staining through assessment of the following parameters: epithelial thickness, collagen fraction, and inflammatory cell count.

### 2.1 Statistical Analysis

The distribution of numerical data was assessed using the Kolmogorov–Smirnov and Shapiro–Wilk tests, which indicated a non‐normal distribution. Consequently, nonparametric tests were employed for analysis. Descriptive statistics were presented as median and interquartile range (IQR). The Kruskal–Wallis test was used to compare differences among groups at each timepoint, followed by Dunn's post hoc test with Bonferroni correction for pairwise comparisons. A p‐value of ≤ 0.05 was considered statistically significant. All statistical analyses were conducted using IBM SPSS Statistics (version 26, IBM Corp., Armonk, NY, USA).

## 3 Results

### 3.1 H&E Staining—Group 1 (CELLBOOSTER Only)

At baseline, immediately after injury and injection, the extracellular matrix appeared loosely organized, with noticeable spaces and a reduced density of cellular components. An inflammatory response was observed, though the pattern of immune cell infiltration differed slightly. Early postinjury reactions, including vascular changes and initial immune activation, were present; however, the structural integrity of the tissue appeared more disrupted.

By the seventh day postinjury, signs of tissue repair became evident, characterized by increased cellular activity and granulation tissue formation. Collagen fibers displayed a disorganized arrangement, and while the epidermal layer was regenerating, it remained underdeveloped. Re‐epithelialization was ongoing, but wound coverage was incomplete. The persistence of inflammatory cells suggested that the resolution of inflammation was still in progress.

By the fourteenth day, the epidermal layer demonstrated substantial improvement, exhibiting a more continuous and structured appearance. However, dermal remodeling appeared to be progressing at a slower rate. Residual inflammatory activity was still present, and collagen fibers remained loosely arranged, indicating that extracellular matrix remodeling was ongoing. Although healing was evident, the restoration of tissue architecture seemed delayed compared to the combination treatment group. Variations in collagen deposition, inflammation resolution, and re‐epithelialization suggested that the additional PCL component may have contributed to a more structured and accelerated healing response.

### 3.2 H&E Staining—Group 2 (PCL Only)

At baseline (immediately postinjury), histological examination revealed disrupted epidermal continuity with a distinct wound gap, exposing the underlying dermis. The dermal layer exhibited significant structural disorganization, with loosely arranged collagen fibers and evidence of hemorrhage. Early inflammatory changes were initiated, though neutrophilic infiltration remained minimal at this stage. No substantial fibroblast proliferation or extracellular matrix (ECM) deposition was observed, indicating the initial phase of wound healing.

By day 7, the wound had progressed into the proliferative phase, characterized by partial epithelialization with migrating basal keratinocytes at the wound margins. The dermis demonstrated dense infiltration of inflammatory cells, predominantly neutrophils and macrophages, actively clearing debris and facilitating tissue repair. Fibroblast proliferation was evident, contributing to the formation of granulation tissue, with immature, loosely arranged collagen fibers. Neoangiogenesis was prominent, ensuring an adequate supply of oxygen and nutrients for ongoing repair. However, persistent edema and extracellular debris indicated that inflammatory activity was still ongoing.

By day 14, the wound had entered the remodeling phase, exhibiting near‐complete epithelialization with a stratified epithelial layer, albeit with some irregularities. Inflammatory cell infiltration had markedly decreased, with macrophages present at lower densities. Fibroblast activity remained high, promoting collagen maturation and structural organization, signifying the transition toward scar tissue formation. Neovascularization persisted but was less prominent, reflecting the stabilization of the vascular network. Residual granulation tissue was gradually replaced by mature connective tissue, indicating advanced wound resolution.

### 3.3 H&E Staining—Group 3 (PCL + CELLBOOSTER)

At baseline, the histological examination of the PCL + cell booster group revealed subtle differences in the dermal architecture, where the extracellular matrix appeared denser with fewer voids, suggesting a more structured preinjury tissue organization. Minimal inflammatory cell infiltration was observed at this stage.

By the 7th day, distinct differences became apparent between experimental groups. The PCL + cell booster group exhibited a more advanced stage of wound healing, as evidenced by a more structured re‐epithelialization process. The epidermis appeared thicker and more stratified, indicating enhanced keratinocyte proliferation. Additionally, the dermal layer displayed a more organized extracellular matrix with reduced inflammatory infiltration, suggesting a faster resolution of inflammation, possibly facilitated by the immunomodulatory effects of the cell booster. Early fibroblast proliferation and collagen deposition were more pronounced, marking an accelerated transition from the inflammatory phase to the proliferative phase.

By the 14th day, the epidermis exhibited further maturation, with well‐defined layers and restored epithelial continuity. The dermis displayed a more uniform extracellular matrix, with a reduction in edema and a more structured collagen fiber arrangement, indicating advanced tissue remodeling. The presence of inflammatory cells had significantly diminished, while fibroblast activity persisted, contributing to the ongoing wound maturation process. This accelerated reduction in inflammation, along with enhanced collagen organization, suggested that the cell booster played a role in improving the healing trajectory, promoting faster tissue regeneration and structural integrity.

### 3.4 H&E Staining—Group 4 (Control Group)

At baseline, histological slides revealed substantial tissue disruption, characterized by irregularly arranged collagen fibers and the absence of an organized epithelial layer. A high infiltration of inflammatory cells, including neutrophils and monocytes, was observed within the wound site. The extracellular matrix appeared loose, with significant structural disorganization, indicating the early inflammatory response. Minimal evidence of new tissue formation was present, and wound margins remained separated, showing no initial signs of re‐epithelialization.

By the 7th day, inflammatory cell infiltration persisted, although early signs of tissue remodeling became evident. An increase in fibroblast proliferation contributed to extracellular matrix deposition. The formation of granulation tissue was observed, with newly formed blood vessels indicating active angiogenesis. While the inflammatory response remained prominent, macrophage activity had increased, signifying a transition from the acute inflammatory phase to the proliferative phase of wound healing. The epidermis remained incomplete, with only partial coverage over the wound area.

By the 14th day, significant advancements in tissue regeneration were noted. The inflammatory cell population had markedly declined, with a decreased presence of neutrophils and an increased number of fibroblasts and macrophages. The extracellular matrix exhibited greater density, with a more structured collagen arrangement. Re‐epithelialization was nearly complete, with a well‐developed epidermal layer covering the wound. Blood vessels were still present, though their prominence had diminished compared to day 7, suggesting a reduction in angiogenic activity as healing progressed. The overall tissue structure appeared more organized, resembling normal skin, indicating the transition to the remodeling phase.

### 3.5 Masson's Trichrome Staining—Group 1 (CELLBOOSTER Only)

At baseline, Masson's trichrome staining highlighted a disorganized extracellular matrix with loosely arranged collagen fibers stained blue. The collagen density appeared sparse, indicating minimal deposition. Scattered cells, potentially inflammatory or fibroblastic in nature, were embedded within the tissue. Elongated, dark‐stained nuclei, indicative of fibroblast presence, were observed. The overall structural integrity lacked mature collagen bundles, consistent with the early phase of tissue repair.

By day 7, a notable increase in collagen deposition was observed, evidenced by more intense blue staining compared to baseline. However, the collagen fibers remained loosely arranged and fragmented. Cellular infiltration was evident, with numerous nuclei interspersed among the collagen matrix, reflecting ongoing inflammation and fibroblast proliferation. The presence of blood vessels suggested angiogenesis as part of the wound healing process. Despite increased collagen deposition, the extracellular matrix remained immature, and connective tissue organization was still in progress.

By day 14, a substantial improvement in collagen fiber density was noted, with the formation of organized and compact collagen bundles. The blue‐stained collagen fibers appeared thicker and more aligned, indicating progressive extracellular matrix remodeling. Cellular infiltration had declined significantly compared to day 7, suggesting a resolution of inflammation and a transition toward tissue remodeling and maturation. Fibroblasts were still present but in lower abundance, indicating that wound healing was approaching resolution. The presence of mature connective tissue suggested improved structural integrity of the wound site.

### 3.6 Masson's Trichrome Staining—Group 2 (PCL Only)

At baseline, the skin tissue exhibited a well‐structured extracellular matrix with densely packed, mature collagen fibers stained deep blue. The collagen was uniformly distributed throughout the dermis, providing structural integrity. Fibroblast presence was minimal, and no significant inflammatory infiltration was detected, indicating a stable and healthy dermal environment. The extracellular matrix remained intact, with a well‐defined organization essential for maintaining normal tissue function.

By day 7, distinct changes in collagen structure and deposition were observed. The collagen fibers appeared fragmented and less dense compared to baseline, with reduced blue staining intensity, indicating the early stages of collagen remodeling. Inflammatory cells were present, signifying active wound healing and immune response. Fibroblast activity increased, contributing to early granulation tissue formation. The dermis exhibited disrupted structural integrity, with an irregular collagen fiber arrangement, indicating an ongoing regenerative process.

By day 14, collagen deposition had substantially improved, with a significant increase in fiber density. The staining intensity was more pronounced, reflecting ongoing collagen synthesis and maturation. Fibroblast activity remained evident, but inflammatory cell infiltration had diminished, signifying a shift from inflammation to tissue remodeling. The extracellular matrix exhibited a more structured collagen arrangement, suggesting progressive wound closure and dermal restoration. Although the tissue structure was not yet fully matured, the increased collagen fiber alignment indicated an advanced stage of wound healing.

### 3.7 Masson's Trichrome Staining—Group 3 (PCL + CELLBOOSTER)

At baseline, the tissue exhibited loosely arranged collagen fibers with minimal organization. The extracellular matrix appeared relatively sparse, with areas showing an open structure. The presence of inflammatory cells indicated an initial response to injury. Fibroblasts were observed, reflecting the early phase of wound healing. The epidermis appeared intact but showed limited remodeling activity.

By day 7, collagen deposition increased, as evidenced by intensified blue staining. Fibroblasts became more prominent, indicating active extracellular matrix synthesis. The tissue displayed moderate organization, with early signs of granulation tissue formation. Some inflammatory cells persisted, signifying ongoing repair. Although the wound area still exhibited irregular structural features, collagen fiber density improved compared to baseline.

By day 14, collagen fibers became more densely packed and aligned, indicating progressive wound healing and tissue remodeling. Inflammatory cell presence declined significantly, suggesting resolution of the inflammatory phase. Fibroblast activity persisted, contributing to extracellular matrix maturation. The epidermis appeared better structured, indicating ongoing re‐epithelialization.

### 3.8 Masson's Trichrome Staining—Group 4 (Control Group)

At baseline, the histological examination revealed that collagen fibers were fragmented and loosely arranged. A high number of inflammatory cells with dark‐stained nuclei were observed, suggesting an active immune response. The extracellular matrix appeared disorganized, reflecting the initial phase of wound healing.

By day 7, the extracellular matrix remained structurally unorganized. A high density of collagen fibers, stained blue, was detected, suggesting the presence of a developing connective tissue structure. However, these fibers appeared disorganized, indicative of an early stage of tissue repair. Scattered inflammatory cells with dark‐stained nuclei persisted, signifying ongoing immune activity. Minimal evidence of new tissue formation was observed at this stage.

By day 14, a more structured arrangement of collagen fibers was evident. The blue‐stained collagen had begun forming organized bundles, suggesting early extracellular matrix remodeling. Fibroblast‐like cells were visible, interspersed within the collagen, indicating active tissue repair. A few inflammatory cells, including small clusters of dark‐stained nuclei, remained present, though at a reduced density, marking a transition toward the remodeling phase of wound healing.

#### 3.8.1 Epithelial Thickness

**FIGURE 1:**
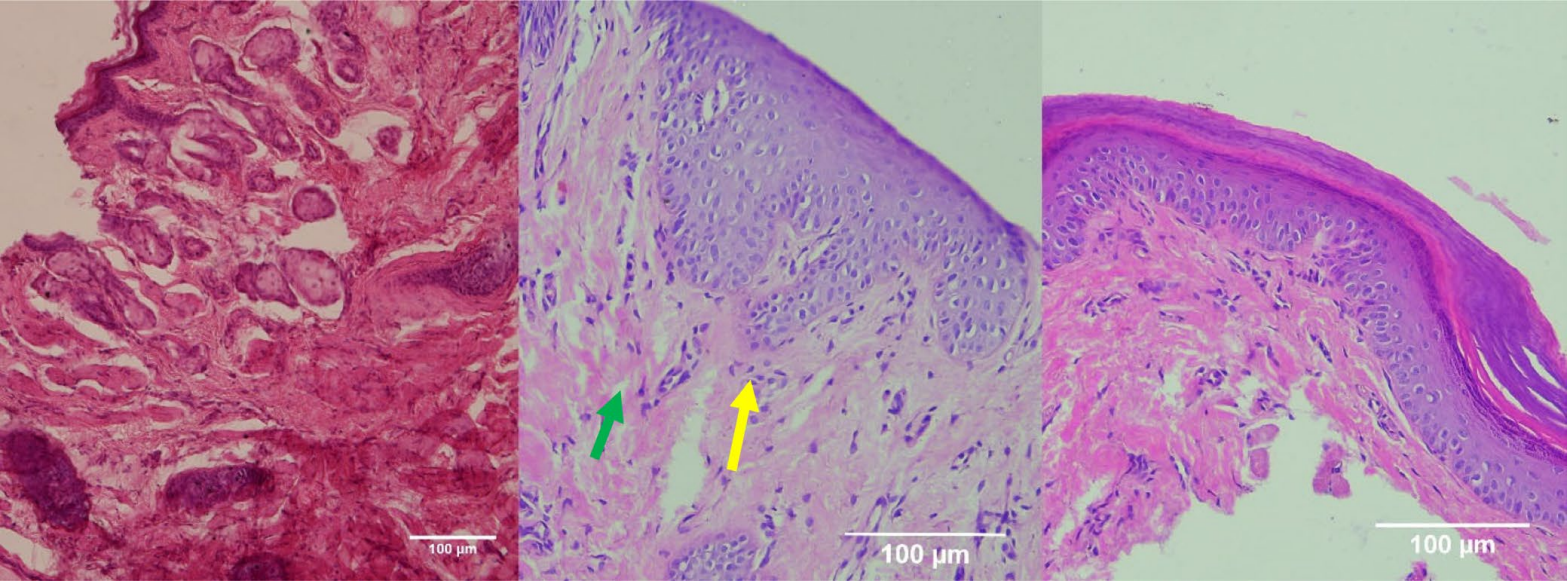
H&E‐stained sections of Group 1 (CELLBOOSTER) at different timepoints. From left to right; Baseline: Wound creation. Day 7: Increased inflammatory cell infiltration (green arrows), early granulation tissue formation (yellow arrows). Day 14: Incomplete reepithelialization, persistent inflammation in some areas.

**FIGURE 2:**
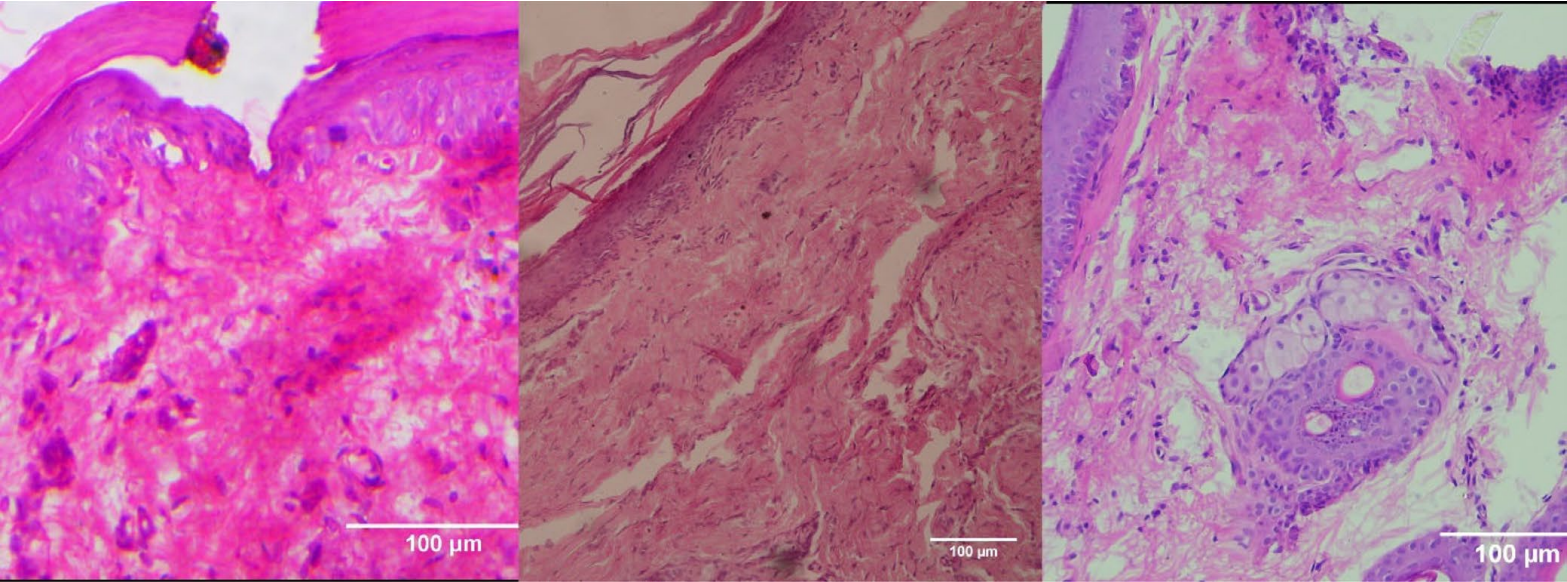
H&E‐stained sections of Group 2 (PCL‐treated group). From left to right; Baseline: Wound creation. Day 7: Increased fibroblast activity and early collagen deposition (red arrows), with moderate inflammatory response. Day 14: Improved epithelial thickness and more structured ECM remodeling, with reduced inflammation compared to the control group.

**FIGURE 3:**
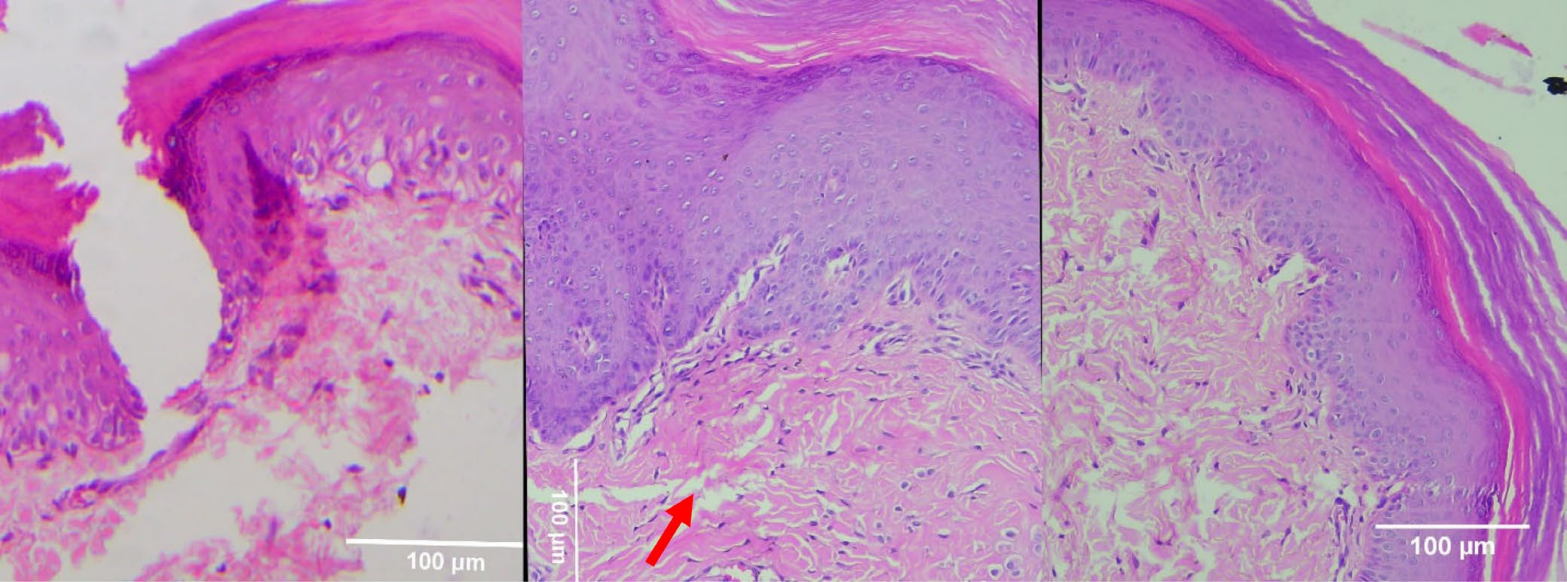
H&E‐stained sections of Group 3 (CELLBOOSTER HA + PCL combination therapy). From left to right: Baseline: Wound induction. Day 7: Reduced inflammation compared to other groups, with early epithelial regeneration and fibroblast infiltration. Day 14: Well‐organized collagen fibers, minimal inflammatory cells, and significant wound closure, indicating enhanced healing.

**FIGURE 4:**
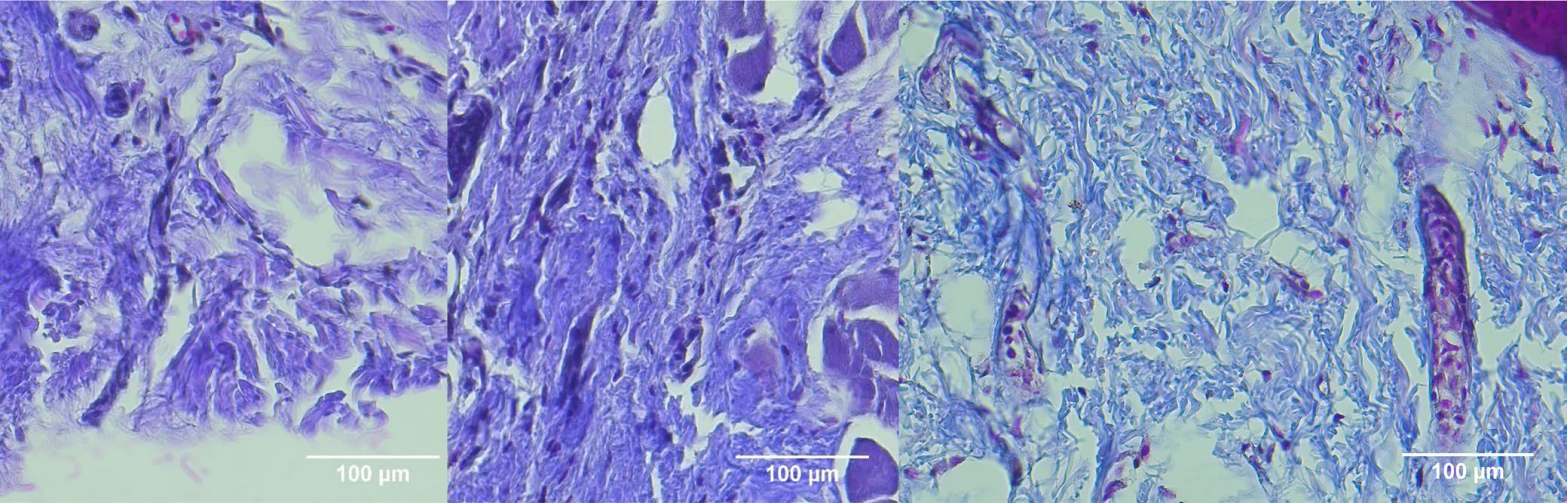
Masson's trichrome (MT) stain of Group 1 (CELLBOOSTER). From left to right; Baseline: Normal ECM structure with no abnormal collagen deposition. Day 7: Early, disorganized collagen formation (blue stain) with ongoing inflammation. Day 14: Less structured collagen fibers and delayed ECM remodeling compared to treated groups.

**FIGURE 5:**
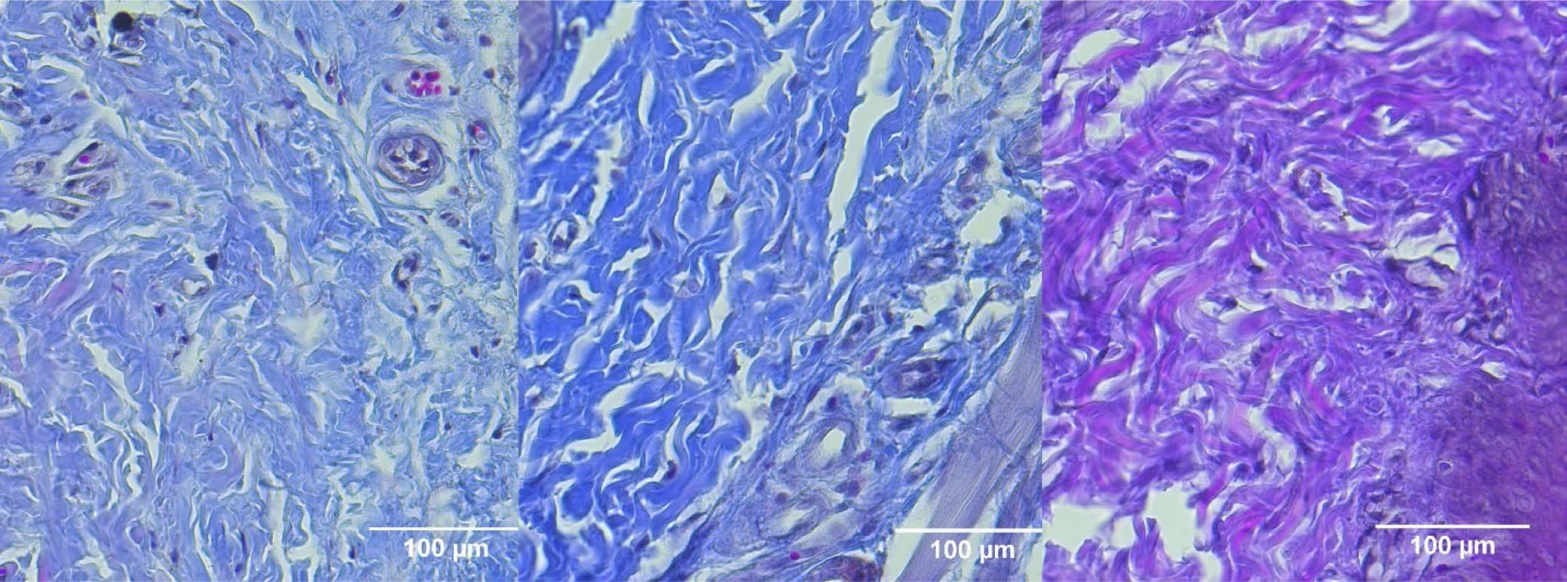
MT stain of Group 2 (PCL‐treated group). From left to right; Baseline: Normal ECM with no significant collagen alterations. Day 7: Increased collagen fiber formation (blue stain), indicating early matrix deposition. Day 14: More organized ECM, with improved collagen fiber alignment, though less structured than the combination therapy group.

**FIGURE 6:**
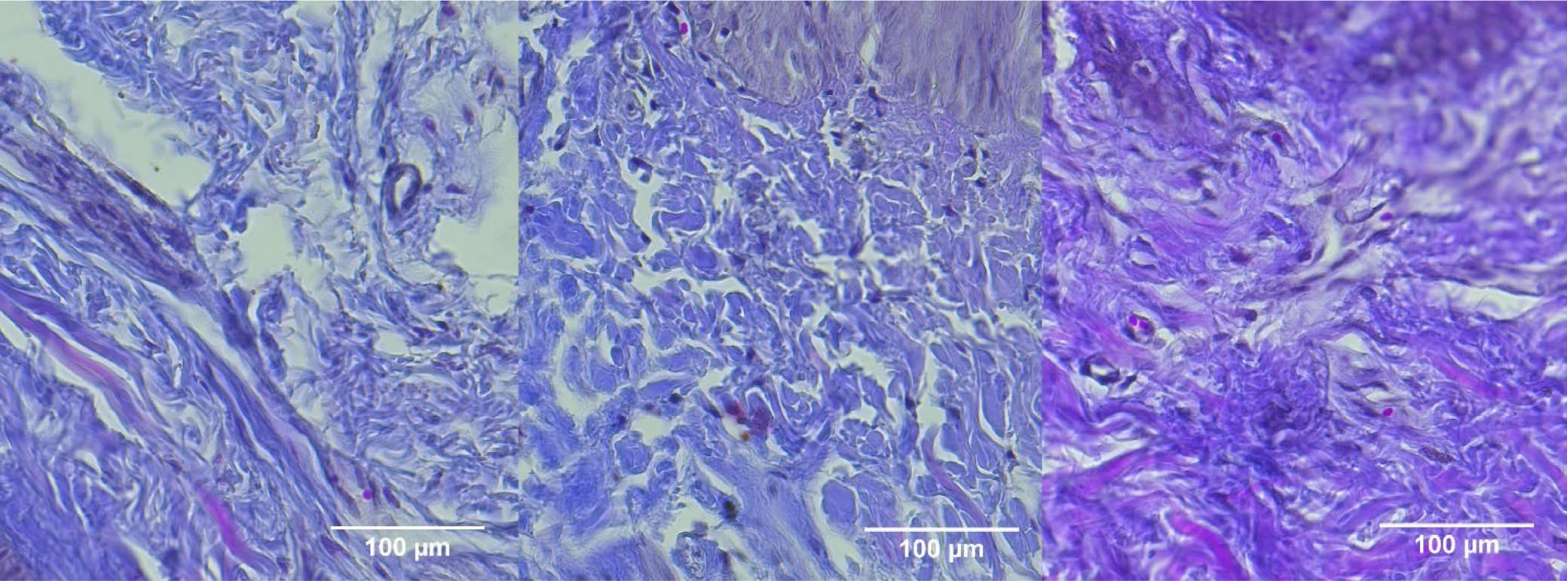
MT stain of Group 3 (HA + PCL combination therapy). From left to right: Baseline: Normal ECM without noticeable collagen deposition changes. Day 7: Early collagen formation, fibroblast infiltration, and reduced inflammation. Day 14: Most structured collagen network among all groups, with minimal inflammation and advanced wound remodeling.

Significant increases in epithelial thickness were observed over time in experimental groups, while the control group showed minimal change. Between‐group comparisons revealed significant differences at multiple timepoints. The highest value of epithelial thickness was recorded on 7th day in the PCL + HA group. Regarding collagen fraction: Groups 1, 2, and 3 exhibited significantly higher collagen deposition over time compared to the control, with substantial increases from baseline to day 14. However, inflammatory cell count: The control group (Group 4) consistently had significantly higher inflammatory cell counts than all other groups across all timepoints, confirming reduced inflammation in treated groups. The PCL + HA group exhibited the lowest amount of inflammatory cells. (Figures 1, 2, 3, 4, 5, 6) (Tables 1, 2, 3, 4, 5, 6).

**TABLE 1:** The mean of epithelial thickness among different groups.

Epithelial thickness			
Group	Baseline	7th day	14th day
Group 1	115.6	145.2	170.8
Group 2	118.2	148.9	175.4
Group 3	120.5	150.3	180.7
Group 4 (control)	95.4	110.7	125.6

**TABLE 2:** The *p* value among different time intervals.

Kruskal–Wallis test results for epithelial thickness		
Timepoint	*p*	Interpretation
Baseline	0.032	Significant
7th day	0.015	Significant
14th day	0.008	Significant

**TABLE 3:** The mean of collagen fraction among different groups.

Collagen fraction			
Group	Baseline	7th day	14th day
Group 1	37.5	52.4	66.9
Group 2	38.9	53.8	68.4
Group 3	40.2	55.7	70.3
Group 4 (control)	20.8	30.2	40.6

**TABLE 4:** The *p* value among different time intervals.

Kruskal–Wallis test results for collagen fraction		
Timepoint	*p*	Interpretation
Baseline	0.041	Significant
7th day	0.019	Significant
14th day	0.006	Significant

**TABLE 5:** The mean of inflammatory cell count among different groups.

Inflammatory cell count			
Group	Baseline	7th day	14th day
Group 1	80.5	70.1	50.4
Group 2	85.2	65.8	45.9
Group 3	88.7	60.4	40.07
Group 4 (control)	110.3	120.6	130.9

**TABLE 6:** The *p* value among different time intervals.

Kruskal–Wallis test results for inflammatory cell count		
Timepoint	*p*	Interpretation
Baseline	0.027	Significant
7th day	0.012	Significant
14th day	0.004	Significant

## 4 Statistical Analysis of Epithelial Thickness, Collagen Fraction, and Inflammatory Cells

## 5 Discussion

Wound healing is a dynamic and highly regulated process that involves a complex interplay of inflammation, cellular proliferation, extracellular matrix (ECM) deposition, and tissue remodeling. Disruptions in any of these phases can lead to impaired healing, chronic wounds, or excessive fibrosis. Various treatment strategies have been explored to enhance the wound healing process, including the use of biostimulatory materials such as hyaluronic acid (HA) and polycaprolactone (PCL) to accelerate tissue regeneration and optimize ECM remodeling [16, 17, 18, 19].

In this study, full‐thickness incisions were made on the dorsal hand region of rats, followed by the injection of either CELL BOOSTER LIFT (HA‐based product), ELLANSE (PCL‐based product), or a combination of both directly into the wound bed. Among the treatment groups, the combined therapy group (CELLBOOSTER HA + PCL) demonstrated the most favorable wound healing outcomes, as evidenced by improved epithelialization, structured collagen deposition, and reduced inflammatory cell infiltration. These findings suggest that the synergistic effects of HA and PCL create an optimal microenvironment for tissue regeneration, potentially surpassing the efficacy of monotherapies.

Although the specific combination therapy used in this study has not been previously reported, our findings align with earlier research evaluating the individual effects of HA and PCL on wound healing. Studies investigating PCL‐based scaffolds have demonstrated their ability to enhance fibroblast activity, increase dermal thickness, and promote collagen deposition, which is consistent with our observations of increased epithelial thickness in the PCL‐treated groups [20, 21]. Similarly, HA has been widely studied for its ability to modulate keratinocyte migration, fibroblast proliferation, and ECM hydration, all of which contribute to enhanced wound repair [22, 23, 24].

HA exerts its wound healing effects through keratinocyte and fibroblast interactions, primarily mediated by CD44 and RHAMM receptors. Activation of CD44‐dependent signaling pathways promotes keratinocyte migration and proliferation, facilitating faster re‐epithelialization. Additionally, HA supports ECM homeostasis by stimulating fibroblast activity, increasing collagen synthesis, and modulating matrix metalloproteinase (MMP) activity, ensuring balanced ECM turnover. HA also plays a role in immune regulation by shifting macrophages toward an anti‐inflammatory M2 phenotype, which helps to control excessive inflammation and fibrosis. The histological findings in our study—particularly the structured ECM remodeling in the HA‐treated groups—align with these known biological mechanisms [25, 26, 27, 28].

Histological analysis revealed that epidermal thickening and granulation tissue formation peaked at the second week following wound induction, consistent with the expected proliferative phase of healing. The combination therapy group exhibited enhanced epithelial thickness, increased collagen density, and more structured granulation tissue, suggesting a well‐organized regenerative process. However, variations in collagen architecture and fibroblast activity were noted between treatment groups, indicating potential differences in tissue responses to the injected biomaterials. By the third week, the wound healing process transitioned into the remodeling phase, where scar tissue typically diminishes, and collagen fibers become more organized. The combination group demonstrated a more structured collagen fiber arrangement and reduced inflammatory infiltrates, suggesting a more controlled remodeling process. While some samples still showed residual fibroblast activity, no evidence of hypertrophic or keloid scarring was observed in any of the experimental groups. The control group, in contrast, exhibited delayed epithelialization and persistent inflammation, further reinforcing the role of HA and PCL in promoting a balanced and efficient wound healing response.

Our findings demonstrate a progressive increase in epidermal thickness during the first 2 weeks of wound healing, with significant tissue remodeling occurring by day 14. Unlike hypertrophic or keloid scarring, which results from excessive fibroblast activation and abnormal collagen deposition, our histological analysis confirmed that the polycaprolactone‐treated group exhibited a balanced healing response. The organized collagen fiber arrangement at day 14 supports the notion that polycaprolactone and hyaluronic acid contribute to improved extracellular matrix remodeling without pathological fibrosis.

Neovascularization was evident in the treated groups, particularly at day 7, with a marked presence of newly formed capillaries in granulation tissue. By day 14, vessel density had stabilized, indicating a transition to the tissue remodeling phase. While angiogenesis was observed histologically, future studies could incorporate quantitative assessments using angiogenic markers such as CD31 and VEGF to further elucidate the molecular mechanisms underlying this process. These findings align with the expected wound healing timeline, emphasizing that our follow‐up period (7 and 14 days) adequately captures the critical phases of inflammation, proliferation, and early remodeling.

To strengthen these findings, future studies should incorporate additional histological markers to quantify tissue remodeling more precisely. Techniques such as immunohistochemical staining for collagen subtypes and fibroblast markers could provide a deeper understanding of the molecular mechanisms involved. Additionally, evaluating the expression of key remodeling regulators, such as matrix metalloproteinases (MMPs) and transforming growth factor‐beta (TGF‐β), could offer insight into the long‐term impact of these injectable scaffolds on wound maturation. Extending the study duration beyond the 14‐day observation period would also help determine whether complete remodeling follows the initial tissue regeneration phase [29, 30, 31].

It was also observed that group 4 (combined injection of polycaprolactone and CELLBOOSTER “HA”) exhibited a pronounced development of collagen fibers as well as increased skin thickness compared to the other groups, accompanied by a low amount of inflammation. In this study, there was no histological or macroscopic evidence of keloid or hypertrophic scar formation in any of the experimental groups but only delayed healing with failure in re‐epithelialization in the control groups at the first week only. While excessive collagen deposition and prolonged fibroblast activity are known contributors to pathological scarring, our findings demonstrated a structured and well‐organized remodeling phase in the combined therapy group. The enhanced collagen deposition observed in treated groups was accompanied by a reduction in inflammatory cell infiltration, suggesting a controlled wound healing response rather than excessive fibrosis. Additionally, polycaprolactone (PCL) and hyaluronic acid (HA) injections promoted balanced extracellular matrix remodeling without signs of aberrant scarring. These findings indicate that the treatment modalities used in this study support optimal wound healing without inducing pathological scar formation. However, further research, including longer follow‐up periods and molecular analyses, could provide deeper insights into the long‐term effects of these biomaterials on tissue remodeling.

Although PCL is widely recognized for its biostimulatory effects on fibroblast activity and ECM remodeling, its influence on angiogenesis remains less explored. In this study, while well‐structured granulation tissue was observed in the PCL‐treated groups, no direct evaluation of neovascularization was performed. Since angiogenesis is best assessed using endothelial markers such as CD31 and VEGF, future studies should incorporate immunohistochemical staining and microvascular density analysis to determine whether PCL actively contributes to vascular network formation during wound healing. The injection of polycaprolactone in this research has demonstrated the capability to increase the skin thickness in the tissue samples, as assessed through histological evaluation and measuring the epithelial thickness. These results were similar to other studies that found that the use of PCL injection led to an increase in dermis thickness, collagen fibers, as well as fibroblast count [32, 33].

At day 7, histological analysis revealed increased epidermal thickness in the treated groups compared to the control, indicating active re‐epithelialization. By day 14, collagen fibers were more organized, and inflammatory cell infiltration was significantly reduced, suggesting early tissue remodeling. No evidence of hypertrophic or keloid scarring was observed at either timepoint. Additionally, the formation of granulation tissue and new capillary structures was evident in the polycaprolactone‐treated group at day 7, signifying enhanced angiogenesis. By day 14, vessel stabilization was observed as part of the wound maturation process.

The presence of bacterial colonization in wounds can significantly impair healing by prolonging inflammation and disrupting tissue regeneration. While this study did not include direct bacterial culture tests, the reduced inflammatory cell infiltration and improved wound closure in the combination therapy group suggest a more favorable microenvironment that may indirectly reduce the risk of bacterial growth. HA is known to exhibit moisture‐retentive properties, which can prevent excessive biofilm formation, while PCL's structured ECM remodeling may enhance barrier function against microbial invasion. Future studies should explore the antibacterial potential of these biomaterials through bacterial inhibition assays, biofilm formation analysis, and immune profiling to determine whether HA and PCL exert direct antimicrobial effects [6, 34, 35, 36].

The findings of this study provide valuable insights into the potential clinical applications of polycaprolactone (PCL) and hyaluronic acid (HA) as injectable biomaterials for enhanced wound healing. Although this study was conducted in a rat model, the biological processes involved in wound healing—such as inflammation, fibroblast activation, collagen remodeling, and re‐epithelialization—are highly conserved across mammalian species, including humans. The observed accelerated epithelialization, increased collagen deposition, and reduced inflammatory cell infiltration in the combination therapy group suggest that HA and PCL could offer therapeutic benefits in human wound healing, particularly in dermatological and reconstructive applications. This may be relevant for:

– Aesthetic procedures (postlaser resurfacing, scar revision).
– Postsurgical wound healing (skin grafts, incisional healing).
– Chronic wound management (diabetic ulcers, pressure sores).

Additionally, the use of injectable biostimulatory materials aligns with current trends in noninvasive regenerative treatments, making them a promising adjunct to existing therapies in cosmetic dermatology, plastic surgery, and tissue engineering. However, translating these findings to human applications requires further research, including clinical trials to assess safety, optimal dosing, and long‐term effects on scar formation and tissue remodeling. Differences in skin structure, immune response, and metabolic processes between rats and humans should also be considered in future studies.

Although this study provides valuable insights into wound healing progression, a more detailed analysis of cellular interactions at the wound edge under higher magnification could further enhance the understanding of epithelial–mesenchymal interactions. This study presents several limitations, including the absence of measurements for elastin formation, epithelial migration, and fibroblast activity. Therefore, it is essential to promote further research that addresses these aspects, along with a comparative analysis of different hybrid injectables. Future studies should incorporate advanced imaging techniques, such as immunohistochemistry for keratinocyte and fibroblast markers, to assess cellular behavior in response to treatment. Moreover, future investigations could benefit from more detailed immune profiling using serial histological sections and immunohistochemical markers for macrophages (CD68), neutrophils (MPO), and T cells (CD3) to assess immune dynamics across different wound regions. Additionally, standardized low‐ to high‐magnification imaging could provide further insight into cellular interactions at various wound depths. These advanced methodologies could further elucidate the impact of polycaprolactone and hyaluronic acid on wound healing mechanisms.

## 6 Conclusion

This study demonstrated that the combined injection of hyaluronic acid rejuvenating complex (HA) and polycaprolactone (PCL) significantly enhanced wound healing by accelerating epithelialization, increasing collagen deposition, and reducing inflammation compared to monotherapies. Histological analysis confirmed that the combination treatment group exhibited the most structured extracellular matrix remodeling, suggesting a synergistic effect of both biomaterials in promoting tissue regeneration. The findings suggest that injectable PCL and HA scaffolds could be a promising approach for optimizing wound healing, particularly in clinical applications requiring enhanced dermal regeneration. However, this study is limited by its observation period, and further research with extended follow‐up is needed to assess long‐term tissue remodeling and scar maturation. Future studies should also incorporate molecular markers for angiogenesis, immune cell dynamics, and collagen subtype differentiation to gain deeper insights into the mechanisms underlying the regenerative effects of these biomaterials. By providing a minimally invasive and biocompatible approach to wound healing, the use of HA and PCL injectables could pave the way for new advancements in regenerative medicine and aesthetic dermatology.

## Author Contributions

N.A.: Created the idea of the research, performed the research, study design, doing all the clinical and experimental works, writing the manuscript, doing the histological analysis, doing the statistical analysis, revising the manuscript, and gathering the data. N.S.: Provided products used in this research and contributed to essential products, assisted in revising the manuscript and assisted in the financials paid for this research as it was self‐funded. G.C.: Contributed essential products, assisted in the financials paid for this research as it was self‐funded. A.G.: Contributed essential products, assisted in the financials paid for this research as it was self‐funded. L.M.S.: Contributed essentialproducts, gave second hand in the historical part and assisted in the financials paid for this research. All authors have read and revised the whole manuscript.

## Ethics Statement

Experimental protocols followed the Guidelines for the Care and Use of Laboratory Animals approved by the Institutional Ethics Committee of Cairo University.

## Conflicts of Interest

The authors declare no conflicts of interest.

## Data Availability

The data that support the findings of this study are available on request from the corresponding author. The data are not publicly available due to privacy or ethical restrictions.
